# Arginase Gene Polymorphism Increases Risk of Diabetic Retinopathy in Type 2 Diabetes Mellitus Patients

**DOI:** 10.3390/jcm10225407

**Published:** 2021-11-19

**Authors:** Monika Buraczynska, Izabela Zakrocka

**Affiliations:** Department of Nephrology, Medical University of Lublin, 20-950 Lublin, Poland; izabela.zakrocka@umlub.pl

**Keywords:** arginase 1, diabetic retinopathy, single nucleotide polymorphism, rs2781666, genotyping

## Abstract

Studies have demonstrated that polymorphic variants of arginase 1 gene (*ARG1*) are involved in human diseases, such as coronary heart disease, hypertension, and diabetes. Our study aimed to investigate the association between *ARG1* rs2781666 single nucleotide polymorphism (SNP) and diabetic retinopathy (DR) in type 2 diabetes (T2DM) patients. Polymorphism was genotyped in 740 T2DM patients and 400 healthy individuals. A significant difference in the genotype distribution was observed between the patients and the controls. The T allele and TT genotype were associated with an increased risk of T2DM (OR 1.4, 95% CI 1.14–1.72, *p* = 0.001 and OR 2.16, 95% CI 1.23–3.80, *p* = 0.007, respectively). When the T2DM subjects were stratified into DR+ and DR− subgroups, the T allele and TT genotype frequencies were significantly higher in the DR+ group compared to the DR− group, demonstrating OR 1.68 (1.33–2.12), *p* < 0.0001 and OR 2.39 (1.36–4.18), *p* = 0.002, respectively. Logistic regression analysis was applied to determine the interaction between the *ARG1* genotypes and other risk factors. Only *ARG1* rs2781666 SNP was a significant risk predictor of DR (*p* = 0.003). In conclusion, this is the first report discussing the effect of *ARG1* polymorphism on the microvascular complications that are associated with diabetes. Our findings demonstrate that *ARG1* rs2781666 SNP is significantly associated with an increased susceptibility to DR in T2DM patients.

## 1. Introduction

Type 2 diabetes mellitus (T2DM) is a chronic, heterogeneous disorder of the glucose metabolism that affects over 450 million people around the world [1]. Diabetic microvascular complications, such as diabetic nephropathy, retinopathy, and neuropathy, are an important cause of morbidity and mortality in T2DM patients [2]. Type 2 diabetes results from the interplay between multiple genetic and environmental risk factors [3]. The conventional risk factors, age, gender, body mass index (BMI), hyperglycemia, and smoking, are insufficient for predicting the development of disease and its complications. Finding additional genes that predispose an individual to type 2 diabetes could provide tools to broaden our knowledge of the complex pathogenesis of diabetes and its vascular complications, resulting in better prevention, diagnosis, and treatment.

Arginase, an important enzyme in the urea cycle, utilizes L-arginine as a substrate to produce urea and ornithine. It is expressed in several cell types, including endothelial cells, macrophages, and vascular smooth muscle cells [4]. Studies have shown that increased arginase activity disturbs nitric oxide synthase (NOS) function, causing the uncoupling of the NOS dimer. Uncoupled NOS produces superoxide instead of nitric oxide (NO), which causes the production of the proinflammatory oxidant peroxynitrite. This may lead to vascular dysfunction in a range of diseases, including diabetes [5,6]. Studies in animal models have reported that of diabetes increases in the levels of arginase contribute to endothelial cell dysfunction [7,8,9].

Two types of mammalian arginase exist: arginase 1 and arginase 2, both of which are encoded by separate genes [10]. Arginase 1 gene (*ARG1*) is located on chromosome 6q23 [11]. It has genomic length of 11.5 kb and consists of eight exons [12]. There are several single nucleotide polymorphisms (SNPs) that are spaced throughout the *ARG1* gene. These polymorphic variants of the *ARG1* gene are involved in human diseases. The rs2781666 G/T SNP, which is located in the 5′ promoter sequence, has been described to be associated with myocardial infarction [13,14], coronary artery disease [15], essential hypertension [16], and diabetes [17]. To the best of our knowledge, there are no data available on the association of *ARG1* gene polymorphisms with diabetic microvascular complications.

The objective of our preliminary case–control study was to analyze the potential association between the *ARG1* rs2781666 SNP (selected on the basis of its position and/or putative functionality) and diabetic retinopathy in T2DM patients.

## 2. Materials and Methods

### 2.1. Subjects

All of the individuals who were involved in this retrospective cross-sectional study were recruited from University Hospital, Medical University of Lublin. The study comprised 740 unrelated T2DM patients (392 males and 348 females) who had been managing a diabetes diagnosis for 10 years or more (mean age 57.2 ± 9.1 years). All of the subjects who were included were Caucasian and of Polish origin.

Diabetes diagnosis was based on the American Diabetes Association criteria for the diagnosis of T2DM [18]. At least one of the following conditions was required for diagnosis: the classic symptoms of hyperglycemia (polyuria, polydipsia, loss of weight), increased plasma glucose levels: fasting > 7 mmol/L or random > 11 mmol/L, and receiving treatment with insulin or oral hypoglycemics. The complete physical examination included plasma fasting glucose, glycated hemoglobin (HbA1c), lipid profile, albumin-to-creatinine ratio (ACR), albumin excretion rate (AER), and body mass index (BMI).

Diabetic retinopathy (DR) was diagnosed by independent ophthalmologists in 445 patients. Of this group, 182 patients presented with concomitant diabetic nephropathy (DN), and those were not analyzed in this study. Of the 263 patients with DR and no DN, 43 had proliferative diabetic retinopathy (PDR) and 220 had non-proliferative diabetic retinopathy (NPDR). All of the patients received a thorough ophthalmological examination that included visual acuity, fundoscopic evaluation, and color fundus photography. The fundoscopic findings were evaluated by a retinal specialist. Retinopathy was evaluated and diagnosed by conforming to the Early Treatment Diabetic Retinopathy Study (ETDRS) criteria. These criteria involve the occurrence of microaneurysms, hemorrhages, intraretinal microvascular abnormalities, cotton wool spots, hard exudates, and new vessels. Patients were categorized into two groups: those with retinopathy (proliferative or non-proliferative) (DR+) and those without retinopathy (DR−). Participants with eye diseases that could manifest as retinal pathological lesions were excluded. Subjects with diagnosed diabetic nephropathy were not included in this study.

The healthy control group involved 400 unrelated volunteers (mean age 57.5 ± 8.1 years). They were mostly blood donors and members of the hospital staff who had recently undergone a health examination. They presented with a normal ECG and no clinical evidence of cardiovascular disease (CVD). Additionally, they did not have a known past history of diabetes, eye disease, and cardiovascular or renal disease. A positive family history of renal or cardiovascular disease in first-degree relatives was a criterion for exclusion.

Prior to participation in the study, a written informed consent was obtained from the patients and the healthy controls, in accordance with principles of the 1964 Declaration of Helsinki. The research protocol of the proposed study was approved by the bioethics committee of Medical University of Lublin (approval on 27 February 2020; code number KE-0254/49/2020).

### 2.2. Genotype Determination

Genomic DNA was extracted from peripheral blood leukocytes (stored at −70 °C) that had been obtained by the standard procedure. The *ARG1* SNP rs2781666 was determined by the amplification of the 294 bp DNA fragment by polymerase chain reaction (PCR). The following primer pairs were used for amplification: forward 5′-CGGAAGGATCTTTAAGGTGCC-3′ and reverse 5′-CCATGTGTCCGATGCAGTTCTG-3′. Genomic DNA (200 ng) was amplified in a 30 μL volume. The initial denaturation at 95 °C for 6 min preceded 35 cycles consisting of denaturation at 95 °C, annealing at 60 °C, and extension at 72 °C (1 min each). The final extension step was at 72 °C for 7 min. The PCR product (10 μL) was digested with five units of Tae I restriction endonuclease (Thermo Fisher Scientific) at 37 °C for 12 h. The resulting DNA fragments were resolved by electrophoresis in 2.5% agarose gel. The length of the fragments was 294 bp for the G allele and 178 bp + 116 bp for the T allele. The genotyping results were validated using blind DNA duplicates (96 samples). The rate of concordance was 100%. Additionally, 20 random samples for each genotype were analyzed by automated sequencing in a CEQ 8000 Genetic Analysis System (Beckman Coulter UK Ltd., High Wycombe, Great Britain).

### 2.3. Statistical Analysis

Statistical analysis for this study was accomplished using SPSS 18.0 for Windows (SPSS, Inc., Chicago, IL, USA). For a comparison of the baseline characteristics between the cases and controls, the normally distributed continuous variables were shown as means ± SD. Categorical variables are shown as numbers and percentages. The Hardy–Weinberg equilibrium was calculated with the chi-square test. The distribution of the allele/genotype frequencies was compared between groups and subgroups utilizing a chi-square test of independence with 2 × 2 contingency and *z* statistics. Continuous and categorical variables were compared using the Mann–Whitney test and Pearson’s χ^2^ test of independence. The odds ratios (OR) with corresponding 95% confidence intervals (CI) were calculated for associations. Post hoc power calculations for the observed associations were conducted using an online power calculator (http://osse.bii.a-star.edu.sg/calculation2.php, accessed on 16 November 2021). Logistic regression analysis was conducted for the assessment of the rs278166 association with DR and the interaction with other risk factors. A two-tailed type I error rate of 5% was regarded as statistically significant.

## 3. Results

The genotype of the rs2781666 SNP in the arginase1 gene was analyzed in 740 T2DM patients and 400 healthy individuals, with a genotyping success rate 100%. The demographic, clinical, and laboratory characteristics of patients and controls are presented in Table 1. Of the 740 patients with T2DM, 263 (35.5%) had diabetic retinopathy. All of the results were compared between this subgroup (DR+) and the 477 T2DM patients who did not present with retinopathy (DR−). The DR patients with concomitant diabetic The information given is correct.nephropathy were not included in this study in an effort to avoid the effects of renal insufficiency on the results. The gender distribution was comparable in patients both with and without DR (53% of males in both subgroups) as was the age at the time when the study was conducted (*p* = 0.257). There was a statistically significant difference in the age at diabetes diagnosis (*p* = 0.043) and diabetes duration (*p* < 0.001). There was also a significant difference in the total cholesterol and triglyceride levels and the BMI between groups. In the comparison of the T2DM patients with the healthy controls, significant differences were seen in age, total cholesterol level, and BMI (*p* < 0.001 for all variables).

The genotyping results are presented in Table 2. The genotype frequencies of rs2296545 SNP were in agreement with the frequencies that were predicted by the Hardy–Weinberg equilibrium in both the T2DM and control groups (*p* = 0.824 and *p* = 0.704, respectively). A statistically significant difference in the polymorphism distribution was found between the T2DM patients and the control group. The minor T allele and TT genotype were significantly associated with the increased risk of T2DM (OR 1.4, 95% CI 1.14–1.72, *p* = 0.001 and OR 2.16, 95% CI 1.23–3.80, *p* = 0.007, respectively). The T2DM subjects were stratified into the DR+ and DR− subgroups for a comparison of the rs2187666 distribution (Table 2). The T allele and TT genotype frequencies were significantly increased in the group of T2DM patients with DR compared to the DR− patients, with OR 1.68 (1.33–2.12), *p* < 0.0001 and OR 2.39 (1.36–4.18), *p* = 0.002, respectively. A post hoc statistical power calculation on the basis of the minor (T) allele frequency indicated a power 74.7% for the comparison between the T2DM patients and the controls and a power of 88.3% for a comparison between the DR+ and DR− subgroups.

Due to a relatively small number of subjects with proliferative DR, we did not conduct the subgroup analysis based on DR type.

Table 3 shows the distribution pattern of the ARG1 rs2781666 polymorphism in the DR+ and DR− patients with regard to dominant, recessive, and codominant models of inheritance. After adjustment for age, sex, BMI, duration of diabetes, and hypertension, the minor T allele in both the CT and TT genotypes was associated with an increased risk of developing DR in all of the models of inheritance. A dose dependent pattern in the effect of T allele on DR development was observed in this genotyping analysis. The OR (95% CI) for homozygote TT versus homozygous non-carrier GG was 2.38 (1.36–4.18), while for the heterozygous carrier GT versus GG, it was 1.91 (1.39–2.64). Multiple logistic regression analysis was then applied to determine the possible interaction between the *ARG1* genotypes and other potential risk factors (Table 4). In this analysis, only *ARG1* rs2781666 SNP was found to be a significant risk predictor of diabetic retinopathy (*p* = 0.003).

## 4. Discussion

Diabetic retinopathy is a frequent, severe diabetic microvascular complication that affects the blood vessels in the retina due to prolonged hyperglycemia. Its exact etiology and pathogenesis have yet to be thoroughly elucidated. The genetic influence in DR was estimated to be as high as 27%, and several genes that are responsible for susceptibility to DR have already been identified [19]. In the current study, we explored the possible association of the rs2781666 single nucleotide polymorphism in the *ARG1* gene with susceptibility to diabetic retinopathy in T2DM patients. The reports on the association of genetic variants in the *ARG1* locus with T2DM are still scarce. To the best of our knowledge, ours is the first study to investigate the association of the *ARG1* gene variant with the microvascular complication of T2DM.

In this preliminary study, we demonstrated that rs2781666 SNP in the *ARG1* locus is significantly associated with type 2 diabetes. The minor T allele and TT genotype (in excess in patient group) increased the risk of T2DM by 1.4 and 2.2-fold, respectively. In the multivariate analysis, this association was independent of other risk factors. This finding is in agreement with the report of Shah et al. [17] who, for the first time, described a significant link of rs2781665 and rs2781666 SNPs in the *ARG1* gene to T2DM. They found increased levels of arginase 1 in patients with diabetes carrying the variant genotypes of both SNPs. Earlier studies also have shown that arginase 1 expression is up-regulated in the coronary arteries of diabetic subjects [5].

To investigate the effect of rs2781666 SNP on susceptibility to DR, we stratified T2DM subjects into DR+ and DR− subgroups. The DR patients with concomitant diabetic nephropathy were not included in this study, which was decided in an effort to avoid the effect of renal insufficiency on the results. The frequencies of the T allele and TT genotype were significantly higher in the group of T2DM patients with DR than in those without DR. The risk of developing DR was 1.7-fold higher for the T allele and 2.4-fold higher for homozygous TT genotype. A post hoc statistical power calculations indicated a power of 74.7% for a comparison between the T2DM patients with the controls and power of 88.3% to detect the association between rs2781666 SNP and DR (in DR+ versus DR− subgroups). The mechanism by which the rs2781666 SNP in *ARG1* gene confers susceptibility to diabetic retinopathy is not clear and needs to be explored. Although extensive studies have demonstrated involvement of arginase in diabetes, its role in retinopathy is not fully understood. Most published studies concentrate on the distribution of the enzymes that are involved in arginine metabolism in ocular structures [20]. The studied polymorphism in the promoter region of the gene can possibly modify ARG1 expression. Arginase 1 is expressed and is functionally active in human endothelial cells, so it is plausible that the dysfunction of the vascular endothelium is engaged in the development of DR. Emerging evidence indicates that arginase is a key regulator of nitric oxide signaling and that it is engaged in the negative regulation of NO production in the macrophages and endothelial cells, and its overexpression is deleterious to endothelial cells [21,22,23]. In an elegant study performed in strptozotocin-induced diabetic mice and using high glucose treated retinal endothelial cells, Patel et al. showed that retinal vascular activation and injury are associated with an increase in arginase expression and activity and a decrease in bioavailable NO formation. Simultaneously, the formation of O_2_^−^ is increased. The authors concluded that arginase is a mediator of diabetic retinopathy, and for the first time, it was mechanistically linked with the increased activity of the arginase to retinal vascular injury in diabetes [24]. The results of previously conducted animal studies show that arginase 1 is associated with endothelial dysfunction in hypertension, aging, diabetes, and ischemia [5,25,26,27]. Diabetic retinopathy is associated with a decrease in the blood flow in the retina in both humans and animals [28,29]. In mouse and rat models, an increase in the expression of arginase 1 that is induced by diabetes was shown to be involved in the high glucose-induced deterioration of retinal blood flow by means of the vascular endothelial dysfunction mechanism. It was clearly shown that the diabetes induced impairment of endothelium-dependent vasodilation responses in the retinal vessels of mice and rats is associated with the activation of arginase and an increase in the production of arginase I. This can be prevented by the deletion of one copy of the arginase gene in mice [8]. The effect of *ARG1* gene polymorphism through endothelial dysfunction has been previously proposed in the studies involving cardiovascular diseases. These studies support the role of arginase 1 in vascular pathophysiology [13,14]. However, since arginase 1 has complex functions that depend on the vascular cell type, whether this hypothesis applies to diabetic retinopathy requires more extensive study in the future. Among other topics, Future studies should also explore what the mechanisms that accountable for arginase overexpression and increased activity in diabetic retina are, or which cells are responsible for the arginase-promoted vascular dysfunction observed in diabetic retinopathy.

The strength of our preliminary study is its large sample size, which had a power of over 80% for the observed association of rs2781666 SNP with DR. However, the results have to be interpreted with caution since the study also has some limitations. It was restricted to one SNP in the *ARG1* locus, the polymorphism that has been reported to be associated with type 2 diabetes. Therefore, we cannot exclude the effect of other polymorphisms at this locus on development of DR. The other limitation of the study is that we did not investigate the functional consequences of rs278166 polymorphism. The arginase levels or its enzymatic activity were not determined in the study subjects.

In conclusion, to the best of our knowledge, this is the first published study analyzing the effect of the *ARG1* gene polymorphism on the microvascular complications of diabetes. Our findings show that the rs2781666 SNP in the *ARG1* gene is significantly associated with increased susceptibility to diabetic retinopathy in T2DM patients. A novel observation of this association as well as its potential clinical significance needs to be confirmed in larger studies as well as in other populations.

## Figures and Tables

**Table 1:** Comparison of clinical and laboratory characteristics of T2DM patients with and without DR.

Variables	Healthy Controls	T2DM Patients	DR+	DR−	*p* Value *
N	400	740	263	477	
Gender (male/female)	205/195	392/348	139/124	253/224	
Age (years)	57.5 ± 8.1	60.2 ± 9.4	59.8 ± 10.3	60.6 ± 8.5	0.257
Age at diabetes diagnosis (years)	NA	43.2 ± 7.6	42.6 ± 7.2	43.8 ± 8.0	**0.043**
Diabetes duration (years)	NA	15.0 ± 9.3	16.6 ± 9.1	13.4 ± 9.7	**<0.001**
Hypertension (%)	0	584 (79)	207 (78.7)	377 (79)	0.923
Diabetic retinopathy (%)	0	263 (35.5)	263 (100)	0	
Total cholesterol (mmol/L)	4.0 ± 0.78	4.82 ± 1.2	4.94 ± 1.4	4.71 ± 0.9	**0.006**
HDL cholesterol (mmol/L)	ND	1.21 ± 0.31	1.19 ± 0.32	1.23 ± 0.29	0.084
Triglyceride (mmol/L)	ND	2.1 ± 0.86	2.3 ± 0.86	1.9 ± 0.66	**<0.001**
HbA_1c_ (%)	ND	8.2 ± 2.5	8.4 ± 2.5	8.0 ± 2.5	0.849
Fasting glucose (mmol/L)	4.61 ± 1.23	8.04 ± 3.2	8.31 ± 3.44	7.85 ± 3.04	0.067
BMI (kg/m^2^)	27.1 ± 4.2	29.8 ± 8.8	30.6 ± 8.3	29.1. ± 9.4	**0.030**

T2DM, type 2 diabetes mellitus; DR+, diabetic retinopathy; DR−, without retinopathy; BMI, body mass index; HbA_1c_, glycated hemoglobin; NA, not applicable. ND, not determined. Data are reported as means ± SD or numbers and percentages (in parentheses). * P calculated for DR+ vs. DR−. Significant *p*-values are indicated in bold. In the comparison between the T2DM and control groups, statistically significant *p*-values were observed in age, total cholesterol, and BMI (*p* < 0.001 for all).

**Table 2:** Genotype and allele distribution of ARG1 rs2781666 polymorphism in patients with T2DM and healthy controls.

Genotypes					MAF	OR (95% CI) ^b^	
	N	GG	GT	TT		T Allele	TT Genotype ^a^
T2DM	740	388 (52.5)	294 (39.5)	58 (8)	0.28	1.40 (1.14–1.72) *p* = 0.001	2.16 (1.23–3.80) *p* = 0.007
T2DM DR+	263	109 (41.5)	126 (48)	28 (10.5)	0.35	1.68 (1.33–2.12) *p* < 0.0001	2.39 (1.36–4.18) *p* = 0.002
T2DM DR−	477	279 (58.5)	168 (35.5)	30 (6)	0.24	Ref. for T2DM DR+	
Controls	400	246 (61.5)	137 (34.2)	17 (4.3)	0.21	Ref. for T2DM	

T2DM, type 2 diabetes mellitus; DR+, diabetic retinopathy; DR−, without retinopathy. Genotype distribution is shown as numbers with percentages in parenthesis. ^a^ Calculated versus CC genotype. Hardy–Weinberg equilibrium: χ^2^ = 0.144, *p* = 0.704 for control group; χ^2^ = 0.049, *p* = 0.824 for T2DM patients. ^b^ Logistic regression was conducted for this association analysis.

**Table 3:** Distribution of the *ARG1* rs2781666 polymorphism according to the model of inheritance.

*ARG1* rs2781666	T2DM DR+	T2DM DR−	OR (95% CI) ^c^	*p* Value
G/T Genotypes	(*n* = 263)	(*n* = 477)		
Codominant model				
GG	109	279	ref	-
GT	126	168	1.91 (1.39–2.64) ^a^	0.0001
TT	28	30	2.38 (1.36–4.18) ^a^	0.0023
Dominant model				
GG	109	279	ref	-
GT + TT	154	198	1.99 (1.46–2.70) ^a^	<0.0001
Recessive model				
GG + GT	235	447	ref	-
TT	28	30	1.77 (1.03–3.04) ^b^	0.0368

*ARG1*, arginase 1 gene. T2DM, type 2 diabetes mellitus. Genotype distribution is shown as numbers. Odds ratio is referred to ^a^ GG homozygote and ^b^ GG+GT genotypes. ^c^ Logistic regression was conducted for this association analysis.

**Table 4:** The results of multivariate logistic regression analysis.

Variable	Odds Ratio	95% CI	*p* Value
Age at study	1.19	0.71–1.38	0.080
Gender	1.26	0.83–1.73	0.092
T2DM duration	1.31	0.74–1.96	0.114
Age of onset	1.22	0.55–2.36	0.652
Hypertension	2.09	0.69–4.14	0.093
BMI	1.12	0.88–1.25	0.325
HbA_1c_	1.14	0.93–1.51	0.084
Total cholesterol	0.96	0.58–1.32	0.754
HDL-cholesterol	1.22	0.83–2.19	0.912
Triglycerides	1.08	0.69–1.53	0.721
T allele *	1.48	1.23–2.02	0.003

T2DM, type 2 diabetes mellitus. * *ARG1* rs2781666 polymorphism. An unconditional model of multiple logistic regression analysis was conducted for interaction between *ARG1* gene polymorphism and other variables.

## Data Availability

The data presented in this study are available upon request from the corresponding author.
